# The Importance of Early Treatment in Middle Ear Disease

**Published:** 1890-08

**Authors:** N. C. Steele

**Affiliations:** Chattanooga, Tenn.


### THE IMPORTANCE OF EARLY TREATMENT IN MIDDLE EAR DISEASE.*

BY N. C. STEELE, M. D., CHATTANOOGA, TENN.

I think that of all the cases of deafness, both partial and complete, 99 per centum result from middle ear disease. And it is my conviction that a large proportion of this deafness is preventable by early treatment.

Perhaps every physician present has heard the old saying, which is as mischievous as it is old: "O, he will outgrow it," referring to a child slightly deaf or with a discharging middle

*Read before the Tennessee Medical Society, April, 1890.

ear. It might be embarrassing to urge a show of hands on the question as to how many of us have given just such advice. In fact, I am not sure but that I should have to show both hands, for fifteen or twenty years ago I may not have seen this matter as I now see it. Such an excuse for ignorance should be relegated to the oblivion of the past, to keep company with "nervousness," "neuralgia," "biliousness," etc., which have on many occasions been the diagnoses in cases not fully understood.

About two hours after I told the Secretary I would write a paper on this subject for this meeting, a friend from a neighboring town was in my office and casually spoke to me of having a child with a running ear which he had thought of bringing to me for treatment, if I thought it of sufficient importance to need attention. He said that it had been discharging for two years and that his physician, whom he had consulted early in the case, had advised him not to try to stop the discharge, as it drained the child's system and was thus good for him. The idea seemed to be that the child's body was full of impurities and that the discharging ear was draining the body of the impurities, as a ditch drains a dirty pond or cess-pool. I suggested that, as two drains are better than one, it might be a wise sanitary measure to punch a hole in the drum-membrane of the other ear and start a drain on that side. The physician referred to is an intelligent man, but he received his medical education many years ago. Of course, I do not vouch for the statement that he gave this remarkable advice as to personal drainage, nor would I think of making a charge of ignorance or negligence against my medical brethren, but it does seem to me that too many physicians are careless about diseases of the middle ear.

Ignorance or indifference regarding the gravity of middle ear diseases, and the importance of their early treatment, is almost universal among the laity; therefore physicians should take advantage of every proper opportunity to impart correct views on these points.

We know that inflammation of the middle ear is common as a complication or sequel of many other diseases, such as typhoid fever, small-pox, pneumonia, scarlet fever, measles, tonsillitis, pharyngitis and rhinitis, and of all the complications and sequel of these diseases, we perhaps pay the least attention to those involving the middle ear. This ought not so to be, for hearing is second in importance to only one other special sense. A diseased middle ear neglected means, too often, deafness, and deafness means one of the most unfortunate physical afflictions which can befall a human being.

The deaf are, as a rule, more miserable themselves, and are more disagreeable to those about them, than are the blind.

A child has earache. The mother pours into the ear a little sweet oil and laudanum, or gives it a dose of soothing syrup to quiet it, and dismisses the matter for the time. This may recur again and again. Possibly she may speak to her physician about it some day when he is in the house to see another member of the family. She asks him if he does not think "Johnny will outgrow his earache." She does not think it amounts to much, and besides she does not like to have "doctors tinkering with the ears ; they are too tender to be doctored." The physician, who may be preoccupied with another case, possibly agrees with her, and the case is thus dismissed. By and by the mother notices that the child seems absent-minded ; he does not reply when spoken to in an ordinary tone, and his teacher thinks him stupid or obstinate. The fact is the child is growing deaf from middle ear disease, which may eventuate in the loss of all useful hearing.

A child has a "rising in the head," as acute inflammation of the middle ear is often denominated. While suffering the agonies of intra-tympanic pressure, the little fellow is the subject of due solicitude on the part of both mother and physician, but so soon as the pent-up mucus or muco-pus breaks through the drum-membrane and discharges externally—"beales," as it is called in some localities—the anxiety of the parents, and sometimes of the doctor, subsides. The child is easy and the ear discharging—"draining the system"—why trouble about it any more ? Do not many such cases get on well enough without treatment ? Yes, but the same is true of typhoid fever, pneumonia, small-pox, scarlet fever, parturition, and so on. Would you advise that all such cases be neglected, trusting solely to the restoring power of nature, or hoping that the patients will outgrow those diseases and conditions? "But why insist on the *early treatment* of disease of the middle ear?" It is scarcely necessary to answer that question. We all recognize in a general way, at least, that as a rule, to which there are few exceptions, the longer pathological conditions continue the more mischief they do and the more difficult they are to remedy. This is certainly true of middle ear disases.

To illustrate. Take a discharging middle ear. Treated early it can be restored almost perfectly to its original condition. Let it be neglected for months and years, and one or more of the following results may confidently be expected: enlargement of the hole in the drum-membrane; anchylosis or total destruction of the small bones of the tympanum; aural polypi, mastoid involvement, with more or less permanent loss of hearing. I could give you sad illustrations from my note-book.

Again, there are great numbers of people who are nearly or completely deaf from chronic non-suppurative inflammation of the middle ear. These persons date the beginning of their deafness back five, ten or perhaps twenty-five years. Many of these cases are beyond the skill of even the ear specialist so far as restoring lost hearing is concerned, and many of them are entirely hopeless. Probably a majority of these persons might have had their hearing at least largely preserved by proper treatment begun early and persevered in. Nearly all of them have an ascertainable history of chronic throat or nose trouble, or of an aching or discharging ear.

Although not coming properly within the purview of this paper to speak of the nature and methods of treatment of diseases of the middle ear, I will say that in nearly every instance they are secondary to other diseases, and perhaps universally there coexists naso-pharyngitis in some form. Therefore to treat middle ear disease effectively, it is necessary to treat the naso-pharynx, and often the whole mucous lining of the pharynx and nose and accessory channels, cavities and organs. To neglect that is to fail in your treatment, especially in chronic cases.

